# Semantic Focusing Allows Fully Automated Single-Layer Slide Scanning of Cervical Cytology Slides

**DOI:** 10.1371/journal.pone.0061441

**Published:** 2013-04-09

**Authors:** Bernd Lahrmann, Nektarios A. Valous, Urs Eisenmann, Nicolas Wentzensen, Niels Grabe

**Affiliations:** 1 Hamamatsu Tissue Imaging and Analysis Center (TIGA), BIOQUANT, University Heidelberg, Heidelberg, Germany; 2 National Center of Tumor Diseases, Medical Oncology, University Hospital Heidelberg University Heidelberg, Heidelberg, Germany; 3 Division of Cancer Epidemiology and Genetics, National Cancer Institute, Bethesda, Maryland, United States of America; 4 Institute of Medical Biometry and Informatics, University Hospital Heidelberg, Heidelberg, Germany; 5 Institute of Pathology, University Hospital Heidelberg, Heidelberg, Germany; University Medical Centre Utrecht, The Netherlands

## Abstract

Liquid-based cytology (LBC) in conjunction with Whole-Slide Imaging (WSI) enables the objective and sensitive and quantitative evaluation of biomarkers in cytology. However, the complex three-dimensional distribution of cells on LBC slides requires manual focusing, long scanning-times, and multi-layer scanning. Here, we present a solution that overcomes these limitations in two steps: first, we make sure that focus points are only set on cells. Secondly, we check the total slide focus quality. From a first analysis we detected that superficial dust can be separated from the cell layer (thin layer of cells on the glass slide) itself. Then we analyzed 2,295 individual focus points from 51 LBC slides stained for p16 and Ki67. Using the number of edges in a focus point image, specific color values and size-inclusion filters, focus points detecting cells could be distinguished from focus points on artifacts (accuracy 98.6%). Sharpness as total focus quality of a virtual LBC slide is computed from 5 sharpness features. We trained a multi-parameter SVM classifier on 1,600 images. On an independent validation set of 3,232 cell images we achieved an accuracy of 94.8% for classifying images as focused. Our results show that single-layer scanning of LBC slides is possible and how it can be achieved. We assembled focus point analysis and sharpness classification into a fully automatic, iterative workflow, free of user intervention, which performs repetitive slide scanning as necessary. On 400 LBC slides we achieved a scanning-time of 13.9±10.1 min with 29.1±15.5 focus points. In summary, the integration of semantic focus information into whole-slide imaging allows automatic high-quality imaging of LBC slides and subsequent biomarker analysis.

## Introduction

Cervical cancer is the second most frequent cancer among women worldwide [1], [2]. Cytology-based cervical cancer screening has led to a substantial reduction of cervical cancer incidence and mortality in many industrialized countries [3]. Despite its success, screening with conventional PAP smears faces several limitations: the single-test sensitivity to detect pre-cancerous stages is about 50–60% [4], and thus has to be repeated frequently to achieve high cumulative sensitivity. Further limitations result from difficulties in standardization, different sample preparation techniques, different cytological classifications, and the high inter- and intra-observer variability of cytology interpretation [5]. Over the last two decades, liquid-based cytology (LBC) has been increasingly used in cervical cytology screening [6], [7]. LBC slides contain less debris and provide clearer cell preparations compared to conventional Pap smears. LBC allows preparing multiple slides from the same sample for biomarker studies. Several biomarkers have been evaluated to improve reproducibility and accuracy for cervical cancer screening. One of the most promising biomarkers is cytological staining for p16/Ki-67. Double staining for p16 and Ki-67 in the same cell highlights HPV-transformed cells and is a marker for cervical precancers. p16/Ki-67 staining is performed on liquid-based cytology (LBC) preparations and evaluated manually [8], [9].

Recently, whole slide imaging (WSI) scanners have become available that are capable of generating full digital microscopic images of glass slides [10], [11]. Multiple WSI scanners are available on the market and have been compared in detail in literature [10]. They are frequently used for digitization of full histological slides [12] or Tissue Microarrays (TMAs) [13]. Accordingly, their focusing technology, being a key feature of WSI scanners, has been developed primarily for histological sections. Principally, WSI should also enable the high throughput analysis of the enormously large batches of cytological cervix samples occurring during screening as has been postulated earlier [9]–[11]. In a previous publication [14], we reported the first implementation of a fully automatic image evaluation system for detecting p16^+^ cells on fully imaged cytological ThinPrep™ slides. But the core problem of applying WSI scanners up till now remained that their focusing technology is not adapted to sparsely populated cytology specimens. For example, in [15], the authors showed that the diagnostic accuracy of virtual slides is comparable to glass slides despite an inherent difficulty of acquiring microscopically well focused virtual slides due to the three-dimensional nature of cytological preparations. Also, another study among cytology technologists [16] reported the principal feasibility of whole-slide imaging cytological slides but described the need for extensive focusing in many z-layers. Thus, from our own experience and the other published studies it became apparent that while working mostly perfectly for histological specimen, in cytology focusing is the key bottleneck hindering.

The problems of focusing a LBC slide can be circumvented by multi-layered (z-stack) scanning. However, multi-layer scanning leads to a multiplication in scanning times by a factor of the number of layers. Furthermore, multi-layering severely complicates manual and automatic image analysis.

We here set out to develop a highly efficient autofocusing approach for LBC slides. Slide scanners like the one used here, first determine a set of unblurred (focused) candidate focus images of the slide at chosen focus points [17]. From the determined z-heights at the focus points, a three-dimensional “focus map” is generated, extrapolating the measured height variations to the full slide. Thereby, too few focus points, inadequately sampling the 3D landscape, will lead to a partially unfocused virtual slide. As the movement of the microscope objective during focusing is relatively slow, too many focus points will in turn significantly increase the total time needed for scanning. The main source of erroneous focus maps are focus points targeting undesired objects like dirt or artifacts within the sample, or dust or streaks located on top of the cover slip. The quality of the focus map is also dependent on the optimal spatial distribution of the focus points. Cell numbers on cytology slides may range from less than 100, to more than 150000 cells and can show varying spatial distribution patterns. Technical problems encountered with LBC preparations were analyzed by Song et al. [18]. The authors described preparative difficulties such as too few cells on the slide, thick preparations, cellular material accumulated in some regions, and blood/debris on the slides.

**Figure 1:**
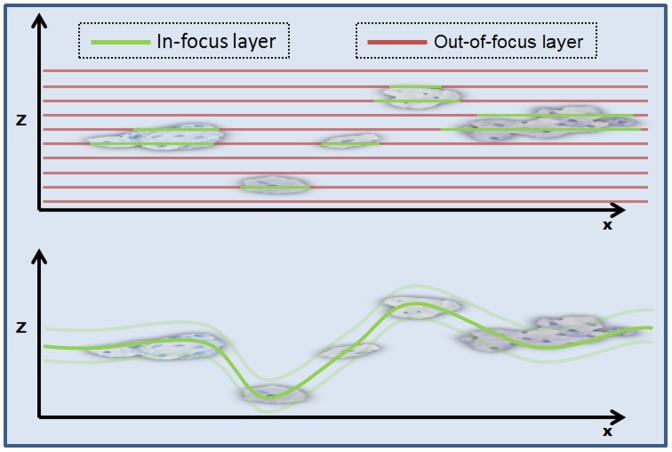
Comparison multi-layer scanning with single-layer scanning. Two cross sections of a LBC slide are shown. The upper one shows the multilayer scanning principle. The lines represent the particular layers. Green line parts represent in-focus regions and red line parts represent the out-of-focus regions. In multilayer scanning, the most parts of the layers are out-of-focus and thereby an unnecessary amount of data is generated. The lower cross section shows the principle of a single-layer scan. A “master-focus layer” (green line) represents the full 3D focus map of the LBC slide. In the optimal case, one focus layer would be sufficient and multi-layering would not be needed anymore or only as a supplement to cover thick cell clusters (transparent green lines).

Concluding, the underlying problem in focusing LBC slides is that semantic information about the scanned sample is missing in general-purpose focusing routines of the slide scanners. General-purpose focusing algorithms are not able to determine whether a focus point is correctly targeting a cell or incorrectly an artifact or whether the whole set of focus points is correctly chosen to capture the essence of the slide. This is because such routines lack any conceptual understanding of cytological liquid based preparation samples. Thus, in this publication we make a first step to incorporate such cytopathological knowledge into the automatic focusing of LBC slides. Such focusing would be optimal if a “master-focus layer” is found, representing the full 3D focus map of the LBC slide. Slide scanning with this master-focus layer could capture each region of the slide in-focus (Figure 1). In the optimal case, one focus layer would be sufficient for scanning and multi-layering would not be needed or only as a supplement to cover thick cell clusters.

To achieve this, we first performed a systematic analysis of the height variations within cytological samples in the *z*-dimension (section 3.1). Then, two image processing algorithms were developed, one cell based, and one slide based. The first one (section 3.2) decides whether a focus point is valid, i.e. detects a cell instead of an artifact. This cell based algorithm implements a semantic auto-focus function neglecting undesired non-cellular objects. The second algorithm determines the total focus quality of a virtual slide from an LBC glass slide. The algorithm thereby yields an objective measurement of a virtual slide's focus quality comparable to a human observer's assessment (3.3). A complete automatic workflow (3.4) was then created to automatically set valid focus points, measure the virtual slide's focus quality and automatically re-scan it in total or partially. In this way, the master-focus layer is iteratively determined. To our best knowledge this is the first reported systematic analysis of whole-slide imaging of LBC slides and the first development of a system, capable of fully automated single-layer focusing of LBC slides.

## Materials and Methods

### Technical setup

LBC slides were digitized using the NanoZoomer HT Scan System (Hamamatsu Photonics, Japan, http://sales.hamamatsu.com/assets/pdf/hpspdf/e_ndp20.pdf) capable of scanning whole slides. The NanoZoomer can scan up to 210 brightfield or multicolor fluorescently stained slides automatically. It is able to digitize the whole slides and it has a Z-stack (or multilayer) capability that allows the focus to move three dimensionally to any part of the slide. The imaging system consists of three 4096×64 pixel TDI-CCD sensors (cell size 8 µm*8 µm) and a 20× objective lens (NA0.75). Flat field correction can be done easily with an empty blank slide. Misaligned lanes can be corrected with a calibration slide provided by Hamamatsu. Standard glass slides were scanned at 20-fold magnification (0.46 µm/pixel). The resulting virtual slides had an averaged compressed file size of 250 MB (JPEG compression, quality factor = 0.9), while uncompressed the file size was about 9 GB. The spatial dimensions of the scanned areas are about 65000×50000 pixels. A direct connection to the scanner control routines was provided by an application programming interface (API). This API provided several methods to call certain control routines, e.g. start scan, start focusing, load slide, unload slide, etc.. This enables a bidirectional communication with the scanner by obtaining live scan information and also sending back control commands to the scanner during the scanning process. The software which controls the main workflow is written mainly in ANSI/ISO C++, and calls MATLAB functions for the image processing tasks during runtime. Scan software and the developed algorithms were running on a personal computer with an Intel Xeon ® E5430 Dual Core, 2.66 GHz, 4 GB RAM with Windows 7 Professional 32 bit operating system.

### Cytological samples and immunostaining

Liquid-based cytology cervical specimens were acquired from women enrolled in a large cross-sectional study of women attending a colposcopy clinic at the University of Oklahoma [19]. Written informed consent was obtained from all women enrolled into the study and Institutional Review Board approval was provided by OUHSC (University of Oklahoma Health Sciences Center) and the US National Cancer Institute. All analyses were conducted on anonymized liquid-based cytological specimens generated using the Thinprep system [20]–[23]. Liquid-based cytology is a method of preparing cytological samples for microscopic examination. Instead of conventional smear preparations, it involves making a suspension of cells from the sample that is used to produce a thin layer of cells on a slide [24]. Slides were generated using the T2000 processor, an automated slide preparation unit. Briefly, the cytological sample is obtained from the transition zone of the uterine cervix. The sample is then dispersed in vial containing a liquid suspension (PreserveCyt®). The vial is then placed under the T2000 processer and the suspension gets centrifuged and passed through a filter to remove obscuring material (blood and mucus) leaving relevant cells on the filter surface. Finally, the cells on the filter are transferred onto a ThinPrep glass slide within a circular area measuring 22 mm in diameter. The slides are then immediately deposited into a fixative bath to be held for staining. All slides were stained using the CINtec® *PLUS* kit (Roche mtm laboratories AG, Heidelberg, Germany) according to the manufacturer's instructions. Briefly, slides were incubated subsequently with two monoclonal antibodies. The first one (E6H4) is directed against p16, followed by a second antibody linked to horseradish peroxidase and detected by adding DAB substrate, generating a brown stain. The second primary antibody is directed against Ki-67, a cellular proliferation marker which is highlighted by a red stain (Fast Red chromogen). All slides were counterstained with Hematoxylin. In this study, 555 LBC slides were analyzed (67 slides for focus point analysis, 88 for slide sharpness analysis and 400 for the analyzing the complete workflow).

## Results

### Z-dimension Analysis of LBC Slides

**Figure 2:**
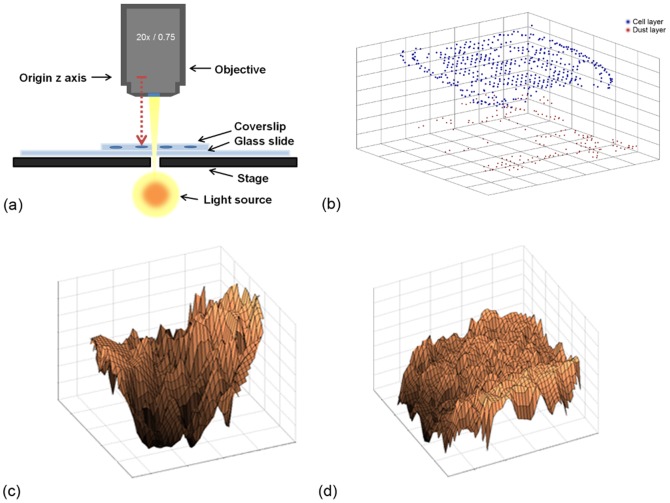
Highly detailed single-slide analysis. (a) A schematic showing the origin of the optical *z*-axis; Red arrow: showing the measured distance from the objective to the measured objects. (b) A 3D graph of the focus points of two different layers which can be found on the slides. The red dots represent points focused on dust which are located on the coverslip. The blue dots are the focus points of the cell layer. The graph looks inverse comparing to the real physical location of the focus points as its origin lies in the lower left corner; (c) a 3D mesh plot of the obtained focus point data only by the cell layer of the slide shows a high degree of heterogeneity within the slides; (d) another example similar to (c) in which smaller variations in the *z* values were observed. The examples in (c) and (d) demonstrate that it is not possible to scan the slides as a planar mono-layer and that there is a high height variation within the slides.

In general, cytological samples do not maintain a perfect planar surface when transferred onto a glass slide in a liquid based preparation. To capture the whole glass slide in an optimal quality, the scanner must be able to detect the height profile of the cells inside the liquid preparation. To obtain information about how many focus points are needed to capture the whole focal variation of a LBC slide, the *z*-dimension ranges of six LBC slides were systematically analyzed. We evaluated how many focus points are necessary to cover the whole z-range variation of the cytological samples. 800 focus points were set on each slide distributed over the whole area covering the cells. These focus points were focused by the scanners' auto focus routine. The scanner then returned the distance relative to a normalized origin of the z-axis located inside the microscope objective. For each focus point, the corresponding image and its coordinates (X, Y and Z) were stored (Figure 2a). All focused objects had a minimum spacing of over 1.9 centimeter to the *z*-axis origin, so we normalized all z-data by this distance. The images at the focus points are acquired with a linear array sensor (4096×96 pixels). The plotted 3D graphs of every slide showed two different layers of focus points on every slide (Figure 2b with a representative graph of an exemplary slide). The first layer (in red) results from focus points from dust on the cover slip and so was incorrectly accepted by the general-purpose auto-focus routine of the scanner. For obtaining the objective height variations of the cell layer, all z-values belonging to measurements of the dust layer were removed. Subsequently, the focus point images were manually inspected and images which were blurred or were focused on artifacts were removed from the dataset. Also, the focus points which were set beyond the borders of the cell circle area were manually removed. The average number of the remaining focus points was 570 per slide. Based on these data, statistical values were extracted in order to obtain information about the focal variation of the focus points like the min, max, and average z values of the particular slides; also the difference between the slides was measured. A mesh was plotted over the surface constructed by the focus point dataset, enabling a visual 3D interpretation of the slides (Figure 2c and 2d). It is apparent that cells inside liquid based LBC slides do not exhibit a planar surface, and that cells with a whole range of different *z* values are located all over the slide. Figure S1 shows a boxplot of the z values of all 6 analyzed slides. The biggest z range within a slide was about 29.5 µm (Table 1). A multilayer scan with a spacing of 2 µm would at least require 15 layers to cover the whole focal variation. The standard deviation of the z values was at least 2.1 µm and at the maximum was 5.7 µm. Scanning one of these slides with one planar layer would necessarily result into out-of-focus regions. The data also shows that the height of the cell layer is different from slide to slide; due to this inter-slide variability an individual focal plane has to be calculated for each particular slide. Also the results show, that the liquid based preparation method does not produce a planar layer of cells but instead a three dimensional gel of probably varying thickness in which the cells are embedded.

**Table 1:** Descriptive statistics of the focus point dataset of the particular slides showing the high variations between the z-values within and between the slides.

Statistics	Cytological samples					
	Slide 1	Slide 2	Slide 3	Slide 4	Slide 5	Slide 6
Nr. of valid focus points	561	423	387	528	323	621
Arithmetic average (µm)	100.9	92.6	127.6	128.1	116.9	69.6
Median (µm)	101.2	93.0	128.1	129.0	116.7	69.8
Standard deviation (µm)	4.0	3.9	5.0	5.7	2.1	3.9
Minimum (µm)	85.9	82.0	113.2	108.4	111.7	59.2
Maximum (µm)	110.4	99.2	137.9	137.9	123.5	77.8
Range (µm)	24.6	17.3	24.7	29.5	11.8	18.6

### Semantic focus point analysis

**Figure 3:**
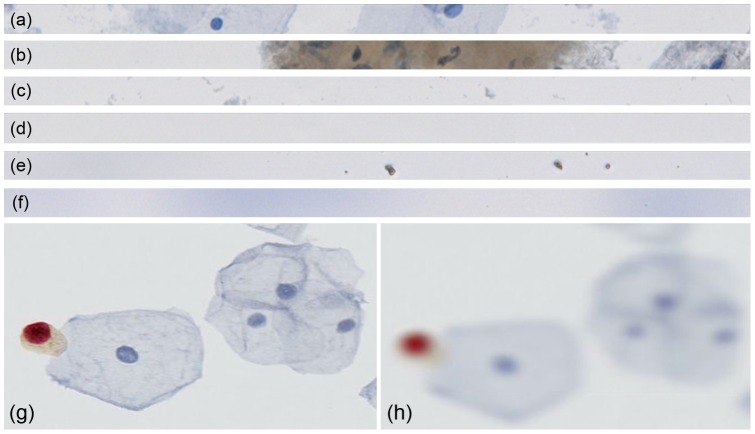
Six different focus point images and comparison of in-focus and out-of-focus images ; (a) a valid image containing in-focus cells. Cells are stained with hematoxilin resulting in blue color; (b) a valid example showing some parts of normal stained cells and cells highlighted with a biomarker conjugated to a brown stain (DAB); (c) an invalid image with only very small objects present; (d) an invalid image with no objects present; (e) an invalid image containing in-focus artifacts; and (f) an example of an invalid out-of-focus image. (g) an example of an in-focus image, and an image which is out-of-focus (h). It is very problematic to obtain relevant image information from (h).

The built-in auto-focus algorithm of the scanner is a contrast-based method which finds the best in-focus image for a given focus point; the inbuilt focus routine calculates the contrast of several images along the optical axis (*z*-plane) of each focus point. The image with the highest contrast is then used for calculating the focus map. However, a contrast-based method in itself is not able to decide whether a focused object is a cell or an artifact. The goal here was to determine and further include criteria enabling the decision whether a focus point image is valid or not. We define a focus point image as only valid if a cell is in focus. If an image is considered not containing cellular material, the corresponding focus point is removed. Figure 3 (a–f) show six different focus point images which were accepted by the built-in auto-focus routine of the scanner although just the above two reflect valid focus points.

From the results of the previous section (3.1) we hypothesized that three filter criteria should be sufficient to judge the validity of an individual focus image. These were maximal object size, the presence of visual object edges and a certain range of frequent color values. We tested then in how far these criteria would apply.

#### Size-filter

Binary objects from the image are obtained by performing a simple thresholding with the average grayscale background intensity from background areas of the slides as a threshold. Subsequently, the number of pixels of each object in the binary focus point image was counted. If none of the objects of the binary image has at least the minimum size of 200 pixels (the size of a typical small nucleus of a superficial cell), the focus point is classified as invalid.

#### Edge-filter

To detect and remove blurred images, an edge detect like the Canny edge (threshold 0.07 and sigma 1.41) detector [25] can be applied to grayscale intensity images. The result of the edge detector is a binary image containing edges of the objects which are present on the images. If an image is blurred, the number of its edges is much smaller compared to an image containing in-focus objects. A simple binary decision classifies the image as invalid if no edges are present.

#### Color-filter

Finally, the color values of the pixels provide valuable information whether they belong to cells. We determined the following image pixel classes which represent all pixel-categories: nuclei, cytoplasm, p16-staining or KI-67 staining. A pixel training dataset encompassing 340 images of nuclei, cytoplasm, background, p16-stained and KI-67 cells (85 images for each class obtained from 10 different slides) was collected. We then manually cropped the corresponding region for each class from the image. Within these regions, 100 pixels were randomly selected, yielding a training dataset of 85,000 pixels per class. On this dataset, an analysis of the individual objects in their respective HSV (hue, saturation, value) channels was carried out. The determined narrow ranges are depicted in Table S1. The focus point image was classified as invalid if less than 10 object pixel belonged to one of these classes. We created a potential classifier encompassing three criteria: images are required to have objects larger than a typical nucleus, at least one edge has to present in the image and 10 pixels have to fall into one of the four valid color categories.

We tested the in-total classification ability of the described criteria with a total data set of 2295 focus point images (containing 190051 objects), obtained from 51 LBC slides. The focus point images are RGB images and had a size of 4096×64 pixels and were acquired in a 20× magnification. The results of the automatic focus point analysis were tested against a manual reference inspection of the focus point images. Valid reference images contained in-focus cells or parts of cells whereas invalid images were out-of-focus, focused on dust, debris or other artifact, or in general were blurred. Table 2 shows the results of the applied algorithm combining edge and color analysis. Both, sensitivity (98.1%) and specificity (99.1%) of the algorithm that classified the focus point images were very high. The positive prediction value, which is the proportion of objects with positive test results that are correctly classified, was 98.9%. The negative prediction value, proportion of objects with a negative test result that are correctly classified as negative was 98.2%.

**Table 2:** Contingency table and overall performance of the focus point analysis of 2295 focus points from 51 LBC slides.

Algorithm classification	Manual Observer Analysis		Predictive value
	Valid	Invalid	
Valid	1086	11	Positive: 98.9%
Invalid	21	1177	Negative: 98.2%
Overall performance of the applied algorithm			
Sensitivity: 98.1%	Specificity: 99.1%	Accuracy: 98.6%	

### Slide Sharpness Analysis

**Figure 4:**
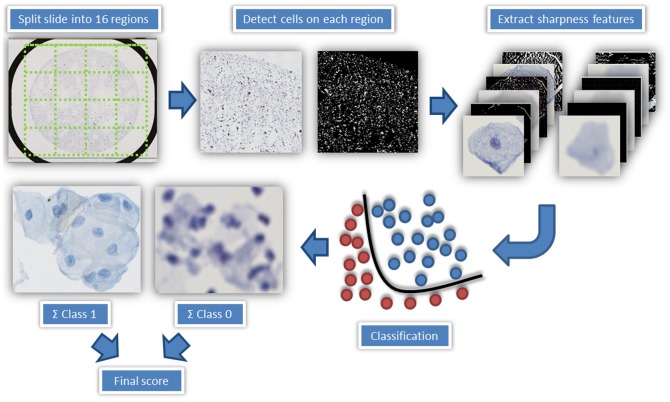
The detailed steps for whole-slide sharpness quantification; At first, the slide is divided into 16 sub-regions. Then, cells are detected by their color values. In total 200 cells are used to quantify the sharpness of each region. For every cell, five sharpness features are computed and a support vector machine (SVM) is used to classify each cell into the in-focus (class 1) or out-of-focus(class 0) category. The percentage of in-focus cells (0–100%) is used to calculate a score for each region, and a combination of these scores is used to represent slide sharpness.

After having obtained a set of valid focus point, the question is whether they are a set in such a way so they accurately sample the three-dimensional height-profile of the LBC glass slide. Figure 3 (g–h) shows a well-focused cell image in contrast to a typical out-of-focus cell image from our data set. Commonly, a slide can exhibit three different quality states which can be determined by a sophisticated classifier: in-focus, partially out-of-focus or completely out-of-focus. To assess this sharpness we developed a special, ThinPrep-Slide dedicated measurement algorithm. The goal of this analysis is to enable an objective assessment on the quality of a slide by a classifier that corresponds to the subjective assessment by a human viewer. Figure 4 shows the concept we propose to perform this measurement.

In the first step, all slides were automatically divided into 16 regions and sample images were extracted automatically from all those regions as follows to determine the overall slide's image quality. The regional division allows a time-saving re-scan of parts of the virtual slide. The scores for the individual regions were later averaged to describe the sharpness of the whole slide. From each region a low resolution overview was extracted from the virtual slide. This overview image was converted into grayscale, and objects were separated from the background using Otsu's segmentation method [26]. The HSV-values of the objects were analyzed to test if the objects are cells. The coordinates of the detected cells were then used to randomly extract up to 200 cell images in the original 20× magnification. An analysis yielded that a higher number of cell images does not increase the accuracy of the slide focus quality analysis (Figure S2 (f)).

To quantitatively measure the sharpness quality of the individual sample images extracted a blind image assessment measurement is required. A blind image assessment technique is independent from any subjective reference standard but requires more sophisticated analysis techniques. We therefore used five different features to quantify the sharpness of a cell image (The features are described in detail in Table S2)). To classify each image a support vector machine (SVM) was used [27]. An SVM maps feature input vectors into a higher dimensional space and constructs an optimal hyper plane separating a set of training data into two groups [28]–[30]. The authors used a Gaussian Radial Basis Function (RBF) kernel with a default scaling factor (sigma) of 1. After initial computation of this hyper plane the SVM can be used as a sharpness classifier. To achieve higher accuracy in the classification, cells which are deemed inappropriate were removed from the training dataset. This includes huge cell clusters (>4 megapixels) and very small cells. A training set constructed containing one class of in-focus cell images and one class of out-of-focus images. The percentage of in-focus cells for each region is then stored and used for calculating an average sharpness score for each slide. In detail, to calculate these sharpness scores, at first the percentage of in-focus cells is determined for each of the 16 regions by examining a maximum number of 200 cells per region. To calculate the average sharpness score for the slides, the percentages of in-focus cells of each region are added and then divided by the total number of regions. The resulting score is then compared with a user defined threshold. If the final score is lower than the threshold, the slide has to be rescanned. These scores basically represent the proportion of in-focus to out-of-focus cells that exist in the slides. Based on the outcome of this analysis, a decision can be made whether to re-scan the whole slide or re-scan specific regions. Another important issue is that this analysis also returns location-specific information of the cells. This is important especially for slides which contain a small number of cells as this information can be used to improve the setting of focus points on these slides.

For the training of the SVM a training set A of 1600 cell images was used. We divided the training set into two classes. The first class was composed of 800 in-focus cell images whereas the other half contained out-of-focus cell images. Cell images were obtained from 63 different slides which were manually scanned. Figure S2 shows the plots of the training dataset of the five different features used for classification. The plots show that it is possible to separate the in-focus cell images from the out-of-focus ones based on these five features (Table S2). For the first 4 features, a linear classifier would be satisfactory to separate the data. For the fifth feature, a nonlinear classifier (Blur metric) had to be applied. The feature vectors of A were the input for the training of the SVM. Feature vectors were also computed from a test set B, which contained 4784 cell images. The test set was obtained from 25 LBC slides which do not belong to the training set.

To determine classification performance, the accuracy, sensitivity and specificity were computed from the test set. Big cell clusters and small cells were not used in the sharpness analysis. Based on these criteria, 1552 cell images were removed from the test set. The remaining 3232 cell images were then classified by the trained SVM. The results of the classification task were manually inspected by two reviewers (The percent agreement regarding focus status for the two observers was: 98%; n = 50 cell images). Table 3 shows the results of the classification task.

**Table 3:** Confusion matrix and overall performance of the classifier used to determine the sharpness of the cell image.

Algorithm Classification	Manual Observer Analysis		Predictive value
	In-focus	Out-of-focus	
In-focus	2207	30	Positive: 98.7%
Out-of-focus	136	859	Negative: 86.3%
**Overall performance of the applied algorithm**			
Sensitivity: 94.2%	Specificity: 95.9%	Accuracy: 94.8%	

### Complete workflow

**Figure 5:**
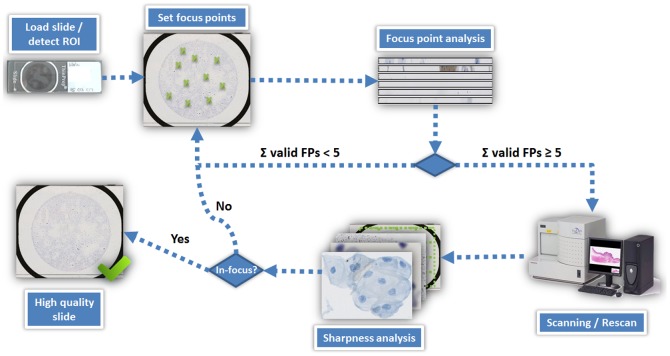
A simplified schematic of the complete workflow for scanning one slide. The slide is loaded and the area to be scanned is detected automatically. Focus points are set and after autofocussing, the focus point images are analyzed. If the number of valid focus point is higher than five, the slide is scanned and its sharpness is analyzed. From the results of sharpness analysis, a decision is made whether to re-scan the slide or not. The slide is re-scanned until the quality is sufficient for further analysis.

Lastly, we evaluated the focusing quality when integrating the previous two algorithms into a single, automated workflow for cytological samples (Figure 5, pseudocode S1). After the slide is loaded, the circular overall region-of-interest on the slide containing all cells is automatically detected. We detect this region by converting a macro image of the slide into a binary image by using Otsu's method (threshold 0.1). ThinPrep slides have black borders surrounding the cell region (cell circle). These borders are very easy to segment by Otsu's method. Furthermore the coordinates of these borders remain nearly constant on every slide. The cell circle is usually placed into the middle of these borders. Therefore, a segmentation of these borders provides the middle point of the cell circle which has a diameter of 22 mm. A set of 12 focus points is then automatically placed on the slide in this area. A smaller number of focus points was in many cases not enough for scanning LBC slides, while a higher number would significantly increase scanning time. After setting the focus points, the built-in auto-focus routine of the scanner commences with the focusing operation and subsequently the focus point analysis begins. If the resulting number of valid focus points is higher than five, the subsequent scanning of the slide is started. If the number of focus points is fewer than five, the number of focus points distributed over the slide is increased, and the scanner repeats the focusing operation. This step is repeated for a maximum number of five times. If the slide is not scanned after the fifth iteration, new focus points are automatically re-set and the whole procedure is re-started. If the slide passes focus point analysis within five iterations, then sharpness analysis is applied on the scanned slide. If the slide is completely out-of-focus, the number of focus points is increased and the slide is re-scanned. If the slide is partially out-of-focus, the out-of-focus regions are re-focused by increasing the number of focus points in these regions and the slide is re-scanned. Consequently, if the slide contains out-of-focus regions, the total sharpness is increased by stepwise improving the sharpness of those particular regions. The re-scanning of the slide is repeated for a maximum number of seven times. Slides which are not in-focus enough for further analysis after these iterations are denoted as “not scannable”.

### Performance analysis of the complete workflow

The complete setup was integrated into the operating software of the scanner to measure performance. This enables the fully automated LBC slide scanning without any user interaction. Slides were determined as focused if their sharpness score was higher than 90%. The integrated workflow was tested with a total set of 400 LBC samples. We measured the time until the slides reached the mark of 90% sharpness in a single layer. Table 4 shows the results of the workflow. An average scan time of 13.9 min per slide was achieved in a single master-layer. The maximum scan time was 55.2 min for one slide and the fastest scan time was 5.7 min. The average number of focus points was 29.1. The maximum number of focus points was 85. The scan iterations which were needed to reach the sharpness criteria of 90% were also measured (Table 5). Nearly 50% of the slides were already successfully scanned after the first slide-scanning iteration. The remaining slides which were not in-focus enough after the first round were refocused on their out-of–focus regions within the next scanning rounds to achieve the required sharpness criteria. The slides which were not completed after the seventh round were automatically aborted (3 slides). These slides contained only a very small number of cells. An exemplary cluster-analysis illustrates the scanning process (Figure S3).

**Table 4:** Scanning duration and number of focus points for the 400 scanned LBC slides.

Statistics	Time (min)
Maximum scan time	55.2
Minimum scan time	5.7
Average scan time	13.9±10.1
Average number of focus points	29.1±15.5

± denotes standard deviation.

**Table 5:** The number of slides finished after each scanning iteration of a total of 400.

Statistics	Data							
Scan round	1	2	3	4	5	6	7	Failed
Number of slides	191	76	64	30	20	10	6	3

## Discussion

Liquid-based cytology (LBC) preparation techniques open new possibilities for a systematic biomarker analysis in cytology. They create clear and rather uniform slides that can facilitate interpretation of Pap-stained and biomarker-enhanced slides. LBC slides are also amenable to high throughput automated analysis. Especially for the detection of rare events on LBC slides, Whole-Slide Imaging (WSI) and subsequent image-processing is of crucial importance for guaranteeing a standardized high quality read out. Unfortunately, digitization of cytological samples is a complex process compared to the routinely used histological tissue samples. Cytological samples have a pronounced three-dimensional profile due to their liquid based preparation and are therefore are harder to capture in sufficient quality. Up till now LBC slides have been digitized by scanning the slides with several layers to cover the whole cell distribution along the z-axis. For example in [31], 15–20 µm is noted as a suitable range for scanning LBC slides, in [15] the slides are scanned at 31.5 µm, in [16] the authors used 20 µm and in [32] the authors conclude that the optimal number of focal planes remains unknown for cytology. Multi-layered imaging substantially hinders the subsequent manual or automatic image processing. There is no guarantee that even multi-layering acquires all necessary objects on the slide. There is no “optimal” number of layers nor of an “optimal” spacing between them [32]. Our results show that multi-layering has so far only been used for LBC slides to circumvent the determination of a “master-focus” layer, which allows the imaging of the far majority of all cells in all regions of the slide in-focus. Therefore we set out to provide the first systematic analysis of ThinPrep slides to determine if such a master-layer can be determined and which proportion of in-focus cells it comprises. As a result of this analysis we stepwise developed an automated imaging procedure for these slides. We then evaluated the resulting overall system and showed, for the first time, that it is a highly effective whole-slide imaging system, capable of the highest quality focusing. Although our approach is based on a specific slide scanner, the Hamamatsu Nanozoomer NDP HT, it can be transferred to all similarly working scanning devices.

To obtain data about the 3D spatial distribution of the cell layer in LBC-prepared cytological samples, the height variation of six different slides were analyzed in a first step. The results showed that cells are indeed arranged in some kind of “mono-layer”, but in a three-dimensional, complex folded height profile. The range of cells along the *z*-axis is up to 29 µm in our examples. Hence, capturing of the corresponding height profile will require a substantial number of focus points to cover the whole height variation of the slide. But scanning with an excessive number of set focus points would result in a substantial loss of time performance. We therefore developed a novel, semantic focus routine, capable of intelligently checking focus points for whether they truly reflect individual cells or cell clusters. We then observed that still this would not be sufficient as it is necessary to place the focus points in such a way that the overall three-dimensional profile of the liquid based preparation is captured. In this process the initial number of focus points deployed is minimal at the beginning but is automatically increased to the necessary extent. Also the location of the placement has to be adapted to allow slide-scanning. During cover slipping of LBC slides, the spatial distribution of the cells is slightly perturbed. This perturbation occurs around points of similar pressure and shows only little variation in a small distance (several millimeters). Therefore, cells that are located in close proximity also have very similar location in the z-axis. Thus, it is possible to find a surface which allows single-layer scanning of the LBC slide. An automated focus analysis must be able to autonomously distinguish between in-focus and out-of-focus cells, and accordingly provide an objective quality measurement for cytological samples. Therefore, we trained a support vector machine with five different features on a training set consisted of over 1600 single cells. The classifier was able to correctly classify in-focus cells with an accuracy of 94.8%. The assembly of the individual steps into a general workflow results in the first system capable of automatically scan liquid based preparation slides.

This was validated with a complete series consisting of 400 LBC slides and only 3 of them were finally not scanned. These slides exhibited only a very small number of cells. In routine diagnostics, such slides would be discarded as inadequate samples. With the implemented approach, we achieved an average total scanning time of 13.9 min. This average time allowed for scanning approximately a hundred slides at one day which is acceptable for high throughput processing. However, a major time consuming part of this approach are the image processing algorithms. Sharpness analysis takes at least 60 sec per slide. If a slide has to be scanned 3 times, then 3 min of the total scan time are used for calculating the sharpness of the slide. Also the scanning-time of the hardware can be expected to improve in future. Thus, several options for accelerating the so far achieved scanning times exist. Already with our current system, cytological samples are scanned within an adequate timeframe in a fully automated manner, without generating too much data. Our results show that indeed a master-focus layer can be determined in LBC slides and that scanning in this layer captures more than 90% of the cells in focus. Thus, our system show that in principle the user does not have to switch manually between multiple *z*-layers and the implementation of image processing algorithms is simpler and more reliable. However, on some slides thick cell clusters may appear. These cell clusters cannot be covered along their entire z-axis by a single layer. Using the determined master-layer as a base layer, with very few additional layers now also these cells can be efficiently captured if requested. An added benefit of the automated sharpness analysis is objectivity compared to manual inspection. All regions of the slide are processed which reduces considerably the probability of “unseen” out-of-focus regions. Concluding, high quality image processing with an effective technique is essential for high quality screening.

Some scanners provide a dynamic focusing option. There, the sample surface profile is tracked while scanning and the focus layer is adapted on the fly. This occurs extremely fast based on physical surface parameters and does not and also cannot comprise any semantic analysis like performed in our work. Our detailed analysis shows that often focusing is impaired by dust or preparation artifacts. In such a case, a dynamic focus would continue to focus in the incorrect z-layer (dust) instead on the cells. Our approach shows for the first time that it is possible to scan LBC slides in a single layer. Current limitations of the approach include high investment costs for the instrument and possibly long scanning times. To obtain information about the reproducibility of the scan quality of the scanner, we scanned one slide 5 times with exactly the same settings and compared the focus quality (98.07%; 97.51%; 97.41%; 97.39%; 97.15%) of the slides. The calculated coefficient of variation (CV) was very low (0.35%) showing that the scanner was very robust by reproducing nearly the same slide quality by scanning with equal settings.

The most exciting result from our work is that we achieved a routine scanning of LBC slides in only one single layer that generated virtual slides of highest quality and suitable for further high throughput analysis. Thus, our system allows fast imaging, and expands the possibilities of automated image based cytological screening.

## Supporting Information

Figure S1 **Detailed analysis of 6 slides.** Boxplot of different *z* values show that a mono-layer of cells is not present in cytological samples. The focal height of the cells is different within a slide, and also among slides.
(TIF)

Figure S2 **Distribution-plots of the five different features used for sharpness analysis obtained from the training set.** Every plot contains 800 in-focus cell images (blue crosses) and 800 out-of-focus cell images (red circles). (a) number of edges, (b) gradient score, (c) difference to sharpened image, (d) difference to blurred image, and (e) Blur metric. Data points with the cross symbol indicate the in-focus class while the diamond symbol indicates the out-of-focus class. The plots demonstrate in practice that it is possible to separate the in-focus from the out-of-focus images based on these features. (f) A plot showing the average sharpness scores of one slide calculated with a different random number of cells per field (x-axis: 10, 50, 100, 150, 200, 350, 500 and 1000). Blue data: every number of cells was tested within 10 runs and the average sharpness scores and the standard deviations are shown. Data showing that increasing the number of cells does not affect the accuracy of the sharpness score significantly after a value of 200 cells per field. Red data: showing that the processing times increases linear with the number of cells processed.
(TIF)

Figure S3 **Iterative gain of total slide sharpness in different batches.** The plots show the sharpness in % of a slide at the current scan iteration. (A) Batch of 20 slides which already reached the 90% after the first or second scan iteration. (B) Batch of 20 slide reaching 90% sharpness after the third or fourth iteration. (C) Slides which needed more than 5 iterations to reach the 90% mark. (D) Of the total of 400 slides, 3 did not reach the 90% mark and were classified as not scannable.
(TIF)

Table S1 **HSV color ranges.** HSV color ranges used to identify object pixel in focus point images.
(PDF)

Table S2 **Sharpness Features.** Detailed description of the five sharpness features used for the classification.
(PDF)

Pseudocode S1 **Pseudo-code for the whole control flow and the total focus quality analysis.**
(PDF)
