# Supplementary figures and images for: Semantic Focusing Allows Fully Automated Single-Layer Slide Scanning of Cervical Cytology Slides
Source: PLoS One. 2013 Apr 9;8(4):e61441. doi: 10.1371/journal.pone.0061441 (PMC3621829; doi:10.1371/journal.pone.0061441)

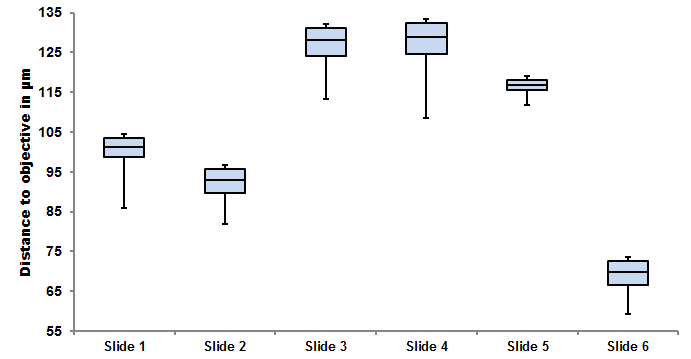

Supplement: Figure S1 — Detailed analysis of 6 slides. Boxplot of different z values show that a mono-layer of cells is not present in cytological samples. The focal height of the cells is different within a slide, and also among slides. (TIF) [file pone.0061441.s001.tif]

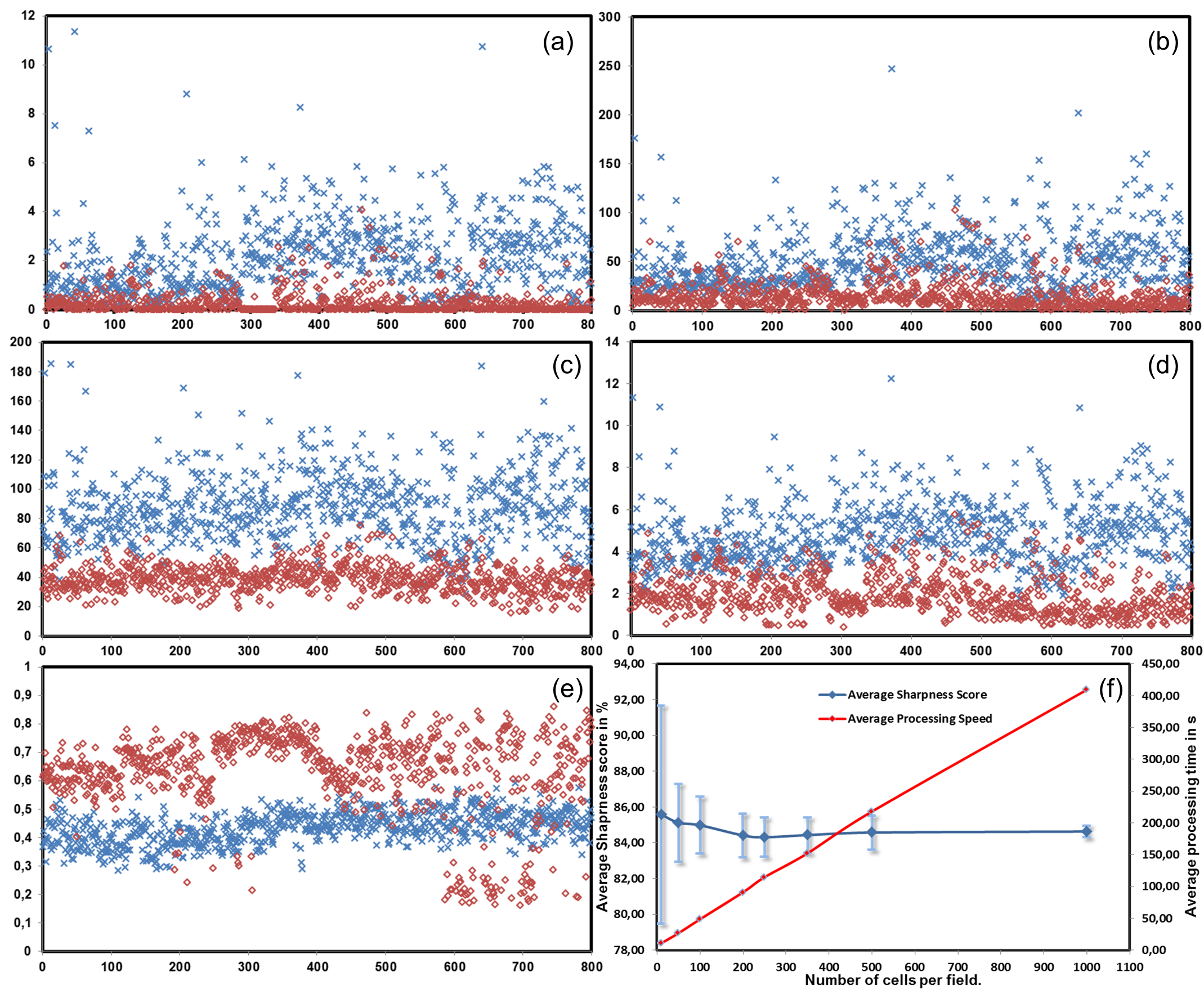

Supplement: Figure S2 — Distribution-plots of the five different features used for sharpness analysis obtained from the training set. Every plot contains 800 in-focus cell images (blue crosses) and 800 out-of-focus cell images (red circles). (a) number of edges, (b) gradient score, (c) difference to sharpened image, (d) difference to blurred image, and (e) Blur metric. Data points with the cross symbol indicate the in-focus class while the diamond symbol indicates the out-of-focus class. The plots demonstrate in practice that it is possible to separate the in-focus from the out-of-focus images based on these features. (f) A plot showing the average sharpness scores of one slide calculated with a different random number of cells per field (x-axis: 10, 50, 100, 150, 200, 350, 500 and 1000). Blue data: every number of cells was tested within 10 runs and the average sharpness scores and the standard deviations are shown. Data showing that increasing the number of cells does not affect the accuracy of the sharpness score significantly after a value of 200 cells per field. Red data: showing that the processing times increases linear with the number of cells processed. (TIF) [file pone.0061441.s002.tif]

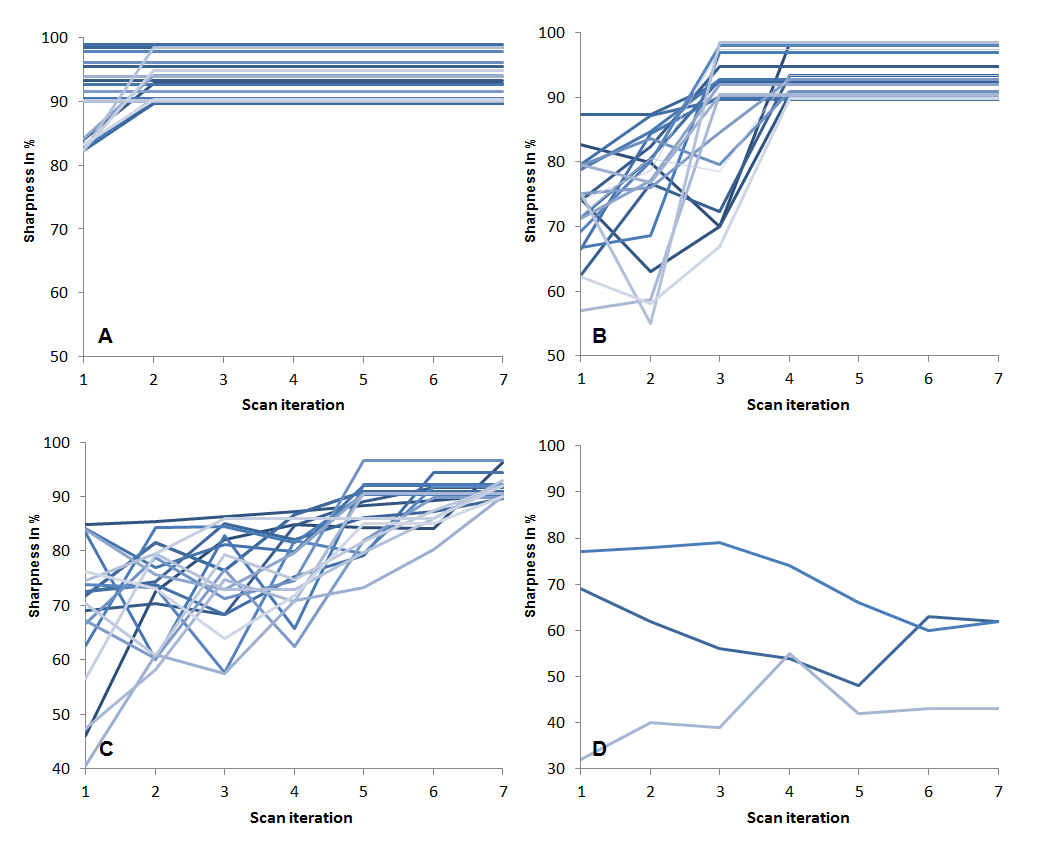

Supplement: Figure S3 — Iterative gain of total slide sharpness in different batches. The plots show the sharpness in % of a slide at the current scan iteration. (A) Batch of 20 slides which already reached the 90% after the first or second scan iteration. (B) Batch of 20 slide reaching 90% sharpness after the third or fourth iteration. (C) Slides which needed more than 5 iterations to reach the 90% mark. (D) Of the total of 400 slides, 3 did not reach the 90% mark and were classified as not scannable. (TIF) [file pone.0061441.s003.tif]
