# Supplementary material for: Semantic Focusing Allows Fully Automated Single-Layer Slide Scanning of Cervical Cytology Slides
Source: PLoS One. 2013 Apr 9;8(4):e61441. doi: 10.1371/journal.pone.0061441 (PMC3621829; doi:10.1371/journal.pone.0061441)
Supplement: Table S1 — HSV color ranges. HSV color ranges used to identify object pixel in focus point images. (PDF) [file pone.0061441.s004.pdf]

Table S1. HSV color ranges used to identify object pixel in focus point images.

Class	Hue	Saturation	Value
Nuclei	100 - 145	60 - 80	10 - 220
Cytoplasm	100 - 145	0 - 40	160 - 240
p16 (DAB staining)	5 - 35	10 - 180	20 - 145
KI-67 (Texas Red)	150 - 185	20 - 65	60 - 170
