# Supplementary material for: Semantic Focusing Allows Fully Automated Single-Layer Slide Scanning of Cervical Cytology Slides
Source: PLoS One. 2013 Apr 9;8(4):e61441. doi: 10.1371/journal.pone.0061441 (PMC3621829; doi:10.1371/journal.pone.0061441)
Supplement: Table S2 — Sharpness Features. Detailed description of the five sharpness features used for the classification. (PDF) [file pone.0061441.s005.pdf]

Table S2. Detailed description of the five sharpness features used for the classification.

**Name**	**Description**
Number of edges	Edges of cell images are detected using a Sobel operator [24]. Number of edges is divided by number of pixels. The result is high for in-focus images containing lots of edges, and low for out-of-focus images.
Gradient score	The numerical gradients in *x* and *y* of the image are computed. They represent intensity changes, which are lower for blurred images compared to in-focus images. A score is calculated by building the sum of both gradient images intensities and dividing it by the number of pixels of the image.
Difference to sharpened image	An unsharp filter is applied to sharpen the images. The difference between result and original is computed. This works well on in-focus images, thus the difference is higher for in-focus images.
Difference to smoothed image	Original images are smoothed with an average filter (disk with a 5 pixel radius) and the difference to original is computed. Average filter affects more the in-focus images, thus the difference is higher.
Blur metric	The Blur metric [25] is computed by the intentional blurring of images. The blurring of in-focus images results in higher grayscale variation compared to lower variation in out-of-focus images. Briefly, the intensity variations between neighboring pixels of the input image are computed. A low-pass filter is then applied on the input image and the variations between the neighboring pixels are also computed on this image. A comparison between these intensity variations of both images allows evaluating the blur annoyance. A high variation between the images means that the original images were sharp wheras a low variation means that the input image was blurred.
