# Supplementary material for: Semantic Focusing Allows Fully Automated Single-Layer Slide Scanning of Cervical Cytology Slides
Source: PLoS One. 2013 Apr 9;8(4):e61441. doi: 10.1371/journal.pone.0061441 (PMC3621829; doi:10.1371/journal.pone.0061441)
Supplement: Pseudocode S1 — Pseudo-code for the whole control flow and the total focus quality analysis. (PDF) [file pone.0061441.s006.pdf]

Pseudo-code for the whole control flow and the total focus quality analysis

```
1: function ControlFlow()
2:     if new slide is loaded then
3:         detect ROI, initialize scan data (slide_file_path, scan magnicifaction etc.)
4:         set Focuspoints → start focusing → focuspoint_analysis(focuspoint images)
5:         if number of valid focuspoints > 5 then
6:             start scanning
7:         else
8:             set more focuspoints
9:             refocus new focuspoints
10:            goto line 5
11:        end if
12:        if scan successful then
13:            f_nf_determineSlideFocusQuality(slide_file_path)
14:            if slide quality is high enough then
15:                done → load next slide
16:            else if slide total unsharp then
17:                add more focus points
18:                start focusing → focuspoint_analysis(focuspoint images)
19:                go to line 5
20:            else if slide partially out of focus then
21:                add focus points in out of focus regions
22:                start focusing → focuspoint_analysis(focuspoint images)
23:                go to line 5
24:            end if
25:        end if
26:    end if
27: end function
```

```
1: function f_nf_ determineSlideFocusQuality(slide_file_path s)
2:     li_overview_img = extract_image_from_slide(s,low_mag)
3:     lo_coordinates = calc_image_statisitic_and_divide_into_16_tiles(overview)
4:     for each image tile do
5:         li_overview_img = extract_image_from_slide(s,low_mag, coordinates)
6:         find cell coordinates of 200 cells in tile
7:         for every found cell do
8:             extract cell image
9:             extract sharpness features
10:            determine sharpness of cell
11:        end for
12:        calculate sharpness score for tile
13:    end for
14:    calculate slides sharpness score
15: end function
```
